# Association between triglyceride glucose index and arterial stiffness in Korean adults

**DOI:** 10.1186/s12933-018-0692-1

**Published:** 2018-03-21

**Authors:** Sang Bae Lee, Chul Woo Ahn, Byoung Kwon Lee, Shinae Kang, Ji Sun Nam, Ji Hong You, Min Jin Kim, Min Kyung Kim, Jong Suk Park

**Affiliations:** 1 Division of Endocrinology, Department of Internal Medicine, Yonsei University College of Medicine, Gangnam Severance Hospital, 211, Eonju-ro, Gangnam-gu, Seoul, South Korea; 2 Severance Institute for Vascular and Metabolic Research, Yonsei University College of Medicine, Seoul, South Korea; 3 Division of Cardiology, Department of Internal Medicine, Yonsei University College of Medicine, Seoul, South Korea; 4 Division of Endocrinology, Department of Internal Medicine, Hallym University Kangdong Sacred Heart Hospital, 150, Seongan-ro, Gangdong-gu, Seoul, South Korea

**Keywords:** Triglyceride glucose index, Arterial stiffness, Pulse wave velocity, Insulin resistance

## Abstract

**Background:**

The triglyceride glucose (TyG) index has been suggested as a simple surrogate marker of insulin resistance. However, there are limited data regarding the association between the TyG index and arterial stiffness in adults. Therefore, we evaluated the relationship between the TyG index and arterial stiffness as measured based on brachial ankle pulse wave velocity (baPWV) in Korean adults.

**Methods:**

A total of 3587 subjects were enrolled in this study. Anthropometric and cardiovascular risk factors were measured. The TyG index was calculated as ln[fasting triglycerides(mg/dl) × fasting glucose(mg/dl)/2], and the insulin resistance index of homeostasis model assessment (HOMA-IR) was estimated. Arterial stiffness was determined by measuring baPWV.

**Results:**

The subjects were stratified into four groups based on the TyG index. There were significant differences in cardiovascular parameters among the groups; the mean baPWV increased significantly with increasing TyG index. According to the logistic regression analysis after adjusting for multiple risk factors, the odds ratio (95% CI) for increased baPWV (> 75th percentile) for the highest and lowest quartiles of the TyG index was 2.92 (1.92–4.44) in men and 1.84 (1.15–2.96) in women, and the odds ratio for increased baPWV for the highest and lowest quartiles of the HOMA-IR was 1.80 (1.17–2.78) in men and 1.46 (1.06–2.47) in women, respectively.

**Conclusion:**

The TyG index is more independently associated with increased arterial stiffness than HOMA-IR in Korean adults.

## Background

Cardiovascular disease (CVD) is a leading cause of death worldwide [1]. In particular, arterial stiffness as measured by brachial–ankle pulse wave velocity (baPWV) has been established as an independent predictor of not only cardiovascular events but also cardiovascular mortality [2].

Insulin resistance (IR) is one of the most important risk factors for CVD [3]. IR is associated with obesity, hypertension, and dyslipidemia, all of which predispose individuals to atherosclerosis and CVD [4]. Indeed, previous studies have demonstrated an independent association between IR and cardiovascular events [5, 6].

Many recent studies have shown that the triglyceride glucose (TyG) index is associated with IR, as assessed by hyperinsulinemic euglycemic clamp testing and HOMA-IR. Thus, the TyG index has been proposed as a reliable and simple surrogate marker of IR [7–10].

Consistent with these data, there is growing evidence to suggest that the TyG index is associated with cardiovascular disease [11–13]. However, few studies have examined the relationship between the TyG index and subclinical atherosclerosis [11, 14, 15]. Moreover, to the best of our knowledge, there has to date only been one study, which was performed in a small population of women, to investigate the relationship between the TyG index and arterial stiffness [16]. Therefore, in the present study, we investigated the relationship between the TyG index and arterial stiffness and compared the data with that of HOMA-IR in Korean adults.

## Methods

### Study population

The study population consisted of 5989 Korean subjects who participated in a comprehensive health examination as part of a self-referred health checkup program at the Gangnam Severance Hospital Health Promotion Center from January 2008 to February 2015. The exclusion criteria of this study were: subjects with elevated triglyceride levels (> 400 mg/dl) and subjects with any history of malignancy, acute inflammatory disease, infectious disease, or renal disease. We also excluded subjects with a history of hypertension, angina, myocardial infarction, cerebrovascular accident, or an ankle–brachial index (ABI) < 0.90. Subjects taking statins or triglyceride-lowering medications (fenofibrate or omega-3) were also excluded. In addition, we excluded men and women with a history of alcohol consumption in excess of 30 and 20 g/day, respectively. After applying the exclusion criteria, a total of 3587 subjects were enrolled in our final analysis. The study protocol was approved by the Institutional Review Board of Yonsei University College of Medicine.

### Clinical characteristics

We measured the height and weight of each study participant and calculated the body mass index (BMI, kg/m^2^). Lifestyle information, personal medical history of acute and chronic illnesses, and medication history were collected using a standard questionnaire. Systolic and diastolic blood pressures (SBP, DBP) were measured by experienced technicians using an automated blood pressure (BP) monitor (HEM-7080IC; Omron Healthcare, Lake Forest, IL, USA) after a 5-min rest period with the patient’s arm placed at heart level. The diagnosis of diabetes mellitus was based on a previous history of diabetes or the ADA’s diagnostic guidelines. Hypertension was defined as systolic and/or diastolic blood pressures > 140/90 mmHg or the current use of an antihypertensive medication. Current smokers were defined as having smoked cigarettes regularly over the previous 6 months.

### Biochemical parameters

Blood samples were obtained from all subjects after 8 h of fasting. Samples were immediately centrifuged, and the serum samples were stored at − 70 °C until analysis. The fasting plasma glucose (FPG), total cholesterol (TC), high-density lipoprotein cholesterol (HDL-C), and triglycerides (TG) were determined using enzymatic methods with a Hitachi 7600–120 automated chemistry analyzer (Hitachi, Tokyo, Japan). Low-density lipoprotein cholesterol (LDL-C) was calculated according to the Friedewald formula. The TyG index was calculated as ln[fasting triglycerides (mg/dl) × fasting glucose (mg/dl)/2] [7]. The fasting serum insulin level was determined by chemiluminescence with an RIA kit (Daiichi, Japan). Insulin resistance was estimated using the homeostasis model assessment of insulin resistance (HOMA-IR) index calculated as fasting insulin (μU/mL) × fasting plasma glucose (mmol/L)/22.5.

### Pulse wave velocity

Arterial stiffness was measured using an automatic plethysmographic instrument (VP-1000; Colin, Komaki, Japan) as previously described [17]. Briefly, electrodes were placed on both wrists, and cuffs were wrapped around both the upper arms and ankles. After simultaneous measurement of blood pressure and waveforms in all four limbs, the time interval between the brachial and ankle waveforms (ΔTba) was determined. The distance between the brachium and the ankle (La − Lb) was estimated automatically according to the subject’s height. After these data were collected, baPWV was calculated using the following equation: baPWV = (La − Lb)/ΔTba (in cm/s). Both baPWV values were measured after allowing the patient to rest in a supine position for at least 5 min. The mean of the right and left baPWV was used as a marker of arterial stiffness.

### Statistical analysis

Continuous variables with normal distributions were expressed as the mean ± standard deviation (SD), whereas continuous variables with skewed distributions were expressed as the medians with interquartile ranges and were log transformed for analysis. Intergroup comparisons were performed using ANOVA. Chi square tests were used to compare categorical variables with percentages. Age-adjusted baPWV means and standard errors were calculated using analysis of covariance (ANCOVA) according to TyG quartiles. The relationships between the baPWV and various clinical parameters were examined using Pearson’s correlation. The odds ratios (OR) and corresponding 95% confidence intervals (CI) for high PWVs were estimated by multivariate logistic regression analysis after adjusting for confounding variables across TyG index and HOMA-IR quartiles. Covariates in the multivariable model, which were chosen for their clinical importance, as well as statistical significance, were age, SBP, BMI, LDL-C, HDL-C, diabetes mellitus, and menopause in women. Because an absolute cut-off value of normal baPWV was not available, high baPWV was arbitrarily defined as a value greater than the cut-off level between the third and fourth quartiles (> 75th percentile), which was 1437.5 cm/s for men and 1411.5 cm/s for women. All statistical analyses were performed using SPSS for Windows 23.0 (SPSS Inc., Chicago, IL, USA). P values less than 0.05 were considered statistically significant.

## Results

### Baseline characteristics

Table 1 shows the clinical and laboratory characteristics of the study population. Subjects were stratified into four groups based on their TyG index levels. Significant differences in metabolic parameters were observed among the groups. SBP, DBP, BMI, FPG, TC, TG, LDL-C, and HOMA-IR were all positively associated with the TyG index, while HDL-C was negatively associated with the TyG index. In addition, smoking status and the prevalence of diabetes were positively associated with TyG index quartiles.

**Table 1:** Clinical characteristics of the study population according to TyG index

	Q1 (lowest)	Q2	Q3	Q4 (highest)	P value
N	897	896	898	896	
Age (years)	49.91 ± 9.83	52.88 ± 8.99	53.30 ± 8.77	52.03 ± 8.68	< 0.01
Sex (M/F)	286/611	433/463	601/297	741/155	< 0.01
SBP (mmHg)	114.60 ± 12.11	117.75 ± 11.94	120.67 ± 10.96	121.99 ± 10.61	< 0.01
DBP (mmHg)	71.42 ± 8.33	73.68 ± 8.03	75.90 ± 7.18	77.14 ± 7.23	< 0.01
BMI (kg/m^2^)	21.82 ± 2.65	22.99 ± 3.02	23.89 ± 2.78	25.04 ± 2.69	< 0.01
FPG (mg/dL)	85.49 ± 8.92	92.54 ± 9.97	96.87 ± 12.59	102.45 ± 18.17	< 0.01
TC (mg/dL)	183.15 ± 33.16	192.48 ± 33.16	193.78 ± 34.35	200.77 ± 35.14	< 0.01
TG (mg/dL)	55 (48–62)	79 (72–88)	111 (99–122)	180 (153–226)	< 0.01
LDL-C (mg/dL)	109.47 ± 29.61	120.93 ± 30.02	123.14 ± 30.93	123.30 ± 31.76	< 0.01
HDL-C (mg/dL)	58.60 ± 12.95	53.26 ± 12.02	47.59 ± 10.88	42.10 ± 8.62	< 0.01
TyG index	7.73 ± 0.21	8.20 ± 0.11	8.57 ± 0.11	9.16 ± 0.28	< 0.01
HOMA-IR	0.60 (0.41–0.91)	0.87 (0.63–1.28)	1.04 (0.77–1.52)	1.37 (0.88–1.97)	< 0.01
Smoking (%)	38 (4.2)	47 (5.2)	80 (8.9)	123 (13.7)	< 0.01
Diabetes (%)	1 (0.1)	3 (0.3)	20 (2.2)	72 (8.0)	< 0.01

Data are the mean ± SD, number (percentage), or median (interquartile range)

*SBP* systolic blood pressure, *DBP* diastolic blood pressure, *BMI* body mass index, *FPG* fasting plasma glucose, *TC* total cholesterol, *TG* triglyceride, *LDL-C* low-density lipoprotein cholesterol, *HDL-C* high-density lipoprotein cholesterol, *TyG* triglyceride glucose, *HOMA-IR* homeostasis model assessment of insulin resistance

**Fig. 1:**
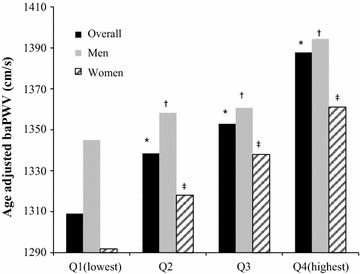
Age-adjusted mean baPWV for the overall population, men, and women (*^, †, ‡^P < 0.01 vs Q1)

We found that baPWV was positively associated with increasing TyG quartiles. Figure 1 shows the age-adjusted mean baPWV according to TyG index quartiles in the overall population, where Q1 = 1308.9, Q2 = 1338.4, Q3 = 1352.9, and Q4 = 1387.7 cm/s (P < 0.01). For men, the age adjusted mean baPWVs of the TyG index quartiles were 1344.9, 1358.2, 1360.7 and 1394.3 cm/s (P < 0.01), while for women the age-adjusted mean baPWVs in the four quartiles were 1291.8, 1318.0, 1337.9 and 1361.1 cm/s, respectively (P < 0.01).

### Correlation between the baPWV and clinical variables

Pearson’s correlation analysis was used to examine the relationship between the baPWV and clinical variables. The baPWV was significantly correlated with age SBP, DBP, BMI, TC, LDL-C, HDL-C, TG/HDL-C, LDL-C/HDL-C, non HDL-C HOMA-IR, and the TyG index (Table 2).

**Table 2:** Correlation between the baPWV and clinical variables

	*r*	*P* value
Age	0.507	< 0.01
SBP	0.355	< 0.01
DBP	0.329	< 0.01
BMI	0.062	< 0.01
TC	0.039	< 0.01
LDL-C	0.047	< 0.01
HDL-C	− 0.085	< 0.01
TG/HDL-C	0.106	< 0.01
LDL-C/HDL-C	0.079	< 0.01
Non HDL-C	0.071	< 0.01
HOMA-IR	0.188	< 0.01
TyG index	0.189	< 0.01

HOMA-IR data were log-transformed because of their non-normal distribution

*PWV* pulse wave velocity, *SBP* systolic blood pressure, *DBP* diastolic blood pressure, *BMI* body mass index, *TC* total cholesterol, *LDL-C* low-density lipoprotein cholesterol, *HDL-C* high-density lipoprotein cholesterol, *TG* triglyceride, *HOMA-IR* homeostasis model assessment of insulin resistance, *TyG* triglyceride glucose

### Logistic regression analysis of TyG index, HOMA-IR and high baPWV

The association between the TyG index and high baPWV was further explored by categorizing the TyG index levels into quartiles and using the first quartile as a reference. Based on age-adjusted multivariable logistic regression analysis, with Q1 set as the reference, the TyG index levels for Q2, Q3, and Q4 were associated with increased ORs for high baPWV in all subjects. Both in men and in women, this relationship remained statistically significant after adjusting for confounding variables. In addition, higher HOMA-IR quartiles were significantly associated with increasing baPWV, and this relationship remained significant even after adjusting for metabolic variables. After adjusting for multiple risk factors, the OR (95% CI) for increased baPWV in the highest HOMA-IR quartile was 1.80 (1.17–2.78) in men and 1.46 (1.06–2.47) in women, whereas the OR of the TyG index in the highest quartile was 2.92 (1.92–4.44) in men and 1.84 (1.15–2.96) in women, respectively (Table 3).

**Table 3:** Odds ratios and 95% confidence intervals for high baPWV according to TyG index and HOMA-IR

	OR (95% CI)				*P* for trend
	Q1 (lowest)	Q2	Q3	Q4 (highest)	
TyG index in men					
Model 1	1.00	1.47 (0.99–2.18)	1.55 (1.07–2.26)	2.31 (1.60–3.33)	< 0.01
Model 2	1.00	1.55 (1.02–2.36)	1.81 (1.20–2.71)	2.92 (1.92–4.44)	< 0.01
TyG index in women					
Model 1	1.00	1.30 (0.85–1.99)	2.00 (1.33–3.01)	2.35 (1.34–4.64)	< 0.01
Model 2	1.00	1.28 (0.80–2.03)	1.56 (0.97–2.52)	1.84 (1.15–2.96)	< 0.01
HOMA-IR in men					
Model 1	1.00	1.24 (0.85–1.83)	1.39 (0.95–2.03)	2.15 (1.48–3.11)	< 0.01
Model 2	1.00	1.19 (0.79–1.78)	1.32 (0.90–1.99)	1.80 (1.17–2.78)	< 0.05
HOMA-IR in women					
Model 1	1.00	1.12 (0.87–1.65)	1.38 (0.97–1.85)	2.13 (1.19–3.79)	< 0.01
Model 2	1.00	1.09 (0.85–1.38)	1.31 (0.88–2.01)	1.46 (1.06–2.47)	< 0.05

Model 1: adjusted for age

Model 2: adjusted for age, SBP, BMI, LDL-C, HDL-C, diabetes mellitus, and menopause (women)

## Discussion

In this study, we identified a significant association between the TyG index and baPWV as a marker of arterial stiffness. To the best of our knowledge, this is the first study to demonstrate such a relationship between the TyG index and arterial stiffness in the general population. Furthermore, our results showed that the TyG index is independently and more strongly associated with increased arterial stiffness compared to HOMA-IR after adjusting for metabolic risk factors.

The TyG index which is a surrogate marker of IR, is known to be associated with metabolic parameters and CVD [12, 13]. Furthermore, in recent studies, it has been proven that the TyG index had some prognostic value to predict type 2 diabetes mellitus in normoglycemic patients [18] and normal-weight patients [19]. Our study showed that most of the metabolic parameters were increased or decreased according to TyG index quartiles, as expected. These results were consistent with those of previous studies [14, 20, 21]. Moreover, we observed a significant correlation between the TyG index and baPWV.

A few reports have investigated whether the TyG index can serve as an independent predictor of subclinical atherosclerosis in both the general population as well as in diabetic subjects [11, 14, 15]. However, to date, only one study has examined the relationship between the TyG index and arterial stiffness. Lambrinoudaki et al. showed an association between the TyG index and arterial stiffness as measured using the PWV between the common carotid artery and common femoral artery [16], but this study was small in size and conducted with only postmenopausal women. However, in the present study, which was conducted in a relatively large number of healthy adults, we demonstrated that the TyG index was independently associated with increased PWV. Compared with HOMA-IR, we found that the TyG index was better at predicting increased arterial stiffness, which was consistent with previous findings showing that the TyG index is more strongly associated with carotid atherosclerosis and the prevalence of CAC than HOMA-IR [14, 15].

Although the mechanism underlying the relationship between TyG index and arterial stiffness has not been fully elucidated, it may be linked to IR. Indeed, previous studies have demonstrated that HOMA-IR, which reflects insulin resistance, is related to PWV [22–24]. Both the TyG index and HOMA-IR are well known representative markers of IR, and they are closely related to each other. However, our findings showed that the TyG index was better associated with arterial stiffness than HOMA-IR, which may be explained by the fact that the two indices reflect different aspects of IR. Specifically, the TyG index reflects IR in the muscle [25, 26], while HOMA-IR reflects IR in the liver [6, 27]. Thus, the TyG index may better reflect peripheral insulin resistance and may be a useful marker of arterial stiffness, including carotid and coronary atherosclerosis [14, 15].

Our study had several limitations. First, this was a cross-sectional observational study. Thus, a causal relationship cannot be established based on the results of this study. Second, the study population consisted of Korean men and women enrolled at a single institution, and the proportions of young adults and pre-menopausal women were relatively small. Thus, the generalizability of the results may be limited. Lastly, we were unable to obtain histories of alcohol consumption and smoking in a quantitative manner, and we could not adjust for nutritional and exercise habits, which can affect blood triglyceride levels.

## Conclusions

We demonstrated a significant association between the TyG index and baPWV as a marker of arterial stiffness in both sexes. Moreover, the TyG index was found to be independently associated with increased arterial stiffness to a greater degree than was HOMA-IR. As a marker of insulin resistance, the TyG index is a simple to calculate, and it appears to be a useful marker of arterial stiffness and thus reflective of cardiovascular risk. Further prospective large-scale studies will be needed to elucidate the exact mechanism of the relationship between the TyG index and arterial stiffness.

## Abbreviations

TyG: triglyceride glucose
HOMA-IR: homeostasis model assessment of insulin resistance
PWV: pulse wave velocity
CVD: cardiovascular disease
IR: insulin resistance
ABI: ankle–brachial index
BMI: body mass index
SBP: systolic blood pressure
DBP: diastolic blood pressure
BP: blood pressure
FPG: fasting plasma glucose
TC: total cholesterol
HDL-C: high-density lipoprotein cholesterol
LDL-C: low-density lipoprotein cholesterol
TG: triglycerides
SD: standard deviation
OR: odds ratio
